# Laboratory Study of the Ultraviolet Radiation Effect on an HDPE Geomembrane

**DOI:** 10.3390/membranes11060390

**Published:** 2021-05-26

**Authors:** Fernando Luiz Lavoie, Marcelo Kobelnik, Clever Aparecido Valentin, Érica Fernanda da Silva Tirelli, Maria de Lurdes Lopes, Jefferson Lins da Silva

**Affiliations:** 1 Department of Civil Engineering, Mauá Institute of Technology, São Caetano do Sul 09580-900, Brazil; erica.tirelli@maua.br; 2 São Carlos School of Engineering, University of São Paulo—USP, São Paulo 13566-590, Brazil; mkobelnik@gmail.com (M.K.); cclever@sc.usp.br (C.A.V.); jefferson@sc.usp.br (J.L.d.S.); 3 CONSTRUCT-GEO, Department of Civil Engineering, University of Porto, 4200-465 Porto, Portugal; lcosta@fe.up.pt

**Keywords:** high-density polyethylene, geomembrane, ultraviolet radiation, durability, thermal analysis

## Abstract

High-density polyethylene (HDPE) geomembranes are polymeric geosynthetic materials usually applied as a liner in environmental facilities due to their good mechanical properties, good welding conditions, and excellent chemical resistance. A geomembrane’s field performance is affected by different conditions and exposures, including ultraviolet radiation, thermal and oxidative exposure, and chemical contact. This article presents an experimental study with a 1.0 mm-thick HDPE virgin geomembrane exposed by the Xenon arc weatherometer for 2160 h and the ultraviolet fluorescent weatherometer for 8760 h to understand the geomembrane’s behavior under ultraviolet exposure. The evaluation was performed using the melt flow index (MFI) test, oxidative-induction time (OIT) tests, tensile test, differential scanning calorimetry (DSC) analysis, and Fourier transform infrared spectroscopy (FTIR) analysis. The sample exposed in the Xenon arc equipment showed a tendency to increase the MFI values during the exposure time. This upward trend may indicate morphological changes in the polymer. The tensile behavior analysis showed a tendency of the sample to lose ductility, without showing brittle behavior. The samples’ OIT test results under both device exposures showed faster antioxidant depletion for the standard OIT test than the high-pressure OIT test. The DSC and FTIR analyses did not demonstrate the polymer’s changes.

## 1. Introduction

Geomembranes are polymeric geosynthetic material used as a barrier in environmental facilities, such as landfills, mining facilities, and leachate ponds. This product is also utilized in waste liquid ponds, water ponds, farm ponds, adduction, and irrigation canals [1,2,3]. High-density polyethylene (HDPE) geomembranes are usually used as liners in environmental construction work due to good mechanical properties, good welding conditions, and excellent chemical resistance [4].

An HDPE geomembrane’s field performance is affected by different conditions and exposures, including ultraviolet radiation, thermal and oxidative exposure, and chemical contact. These exposures can change the polyethylene’s morphology, causing a reaction between the polymer chain with the oxygen molecule. Accelerated laboratory tests and exhumed sample evaluations indicate essential responses for the durability issue [5,6,7].

The synergy between ultraviolet (UV) radiation and thermal exposure leads to the geomembrane’s lifetime [8,9]. The wavelength of solar radiation extends from the infrared (higher than 700 nm), through the visible spectrum (400 to 700 nm), and into the ultraviolet (lower than 400 nm). A series of free radical reactions control the polyolefin’s degradation. Polymer chain scission and polymer property degradation can occur when UV radiation reaches the geomembrane surface [10]. To protect the polymer from UV radiation, carbon black is added to the resin during the manufacturing process, about 2 to 3% in mass, typically with a particle size of 22–25 nm [2,10].

There are different methods to study the ultraviolet radiation effects on HDPE geomembranes, as the outside natural radiation exposure demands long-term evaluation, and by the laboratory, using weatherometers [10]. The evaluation of ultraviolet radiation on HDPE geomembranes at the laboratory can be proceeded by different weatherometers: Xenon arc or an ultraviolet fluorescent tube device. Xenon arc weatherometer use in the geosynthetics field is remote due to the high cost involved [11].

Sahu et al. [12] evaluated the influence of ultraviolet light on the thermal properties of HDPE for different carbon black concentrations from 1 to 3%. The authors used an ultraviolet fluorescent weatherometer for a cycle of 192 h. The Fourier transform infrared spectroscopy (FTIR) and differential scanning calorimetry (DSC) were used to analyze the HDPE behavior before and after UV exposure. The analysis confirms that the carbon black was evenly dispersed without degradation in the HDPE matrix.

Jiang et al. [13] investigated an HDPE resin after 800 h of exposure in a Xenon arc lamp device. Antioxidants and a UV absorber were added to understand their influences on ultraviolet resistance. The authors performed FTIR, DSC, and TG (thermogravimetric) analyses and the mechanical test. The results showed which UV absorbers presented better results than the antioxidants. The samples with UV absorbers added maintained their mechanical properties even after 600 h of exposure.

The field study provided by Reis et al. [14] evaluated five 2.0 mm-thick high-density polyethylene geomembrane samples exposed in 8 different places in Portugal over 12 years. The samples presented slight variations in the tensile properties, increasing elongation properties, which was not explained by the authors. Locations with higher UV indexes suggest an impact on tensile properties and antioxidant depletion.

The changes in the structural properties of high-density polyethylene film exposed to UV-B radiation were studied by Martínez-Romo et al. [15]. The crystalline phase fraction of high-density polyethylene film increased further after 30 days of exposure to UV-B radiation, affecting the physical properties such as stiffness, dissolution resistance, and dimensional stability. The results showed which sample had a high oxidation degree.

Mendes et al. [16] analyzed HDPE samples with and without additives exposed to weathering in Rio de Janeiro, Brazil, up to 4000 h. The additives utilized were a phenol type antioxidant and two HALS-type light stabilizers. The authors used FTIR analysis, DSC analysis to determine the crystallinity, mechanical tests, and the variation of the sample’s molecular weight was evaluated by gel permeation chromatography. The results showed decreased elongation at break for the sample without additives, demonstrating the almost total loss in ductility. The increase in crystallinity and reduction of molecular weight for the sample without additives was still verified. The sample stabilized with the additives remained constant for the exposure time, demonstrating the additives’ effectiveness.

Qureshi et al. [17] evaluated a linear low-density polyethylene (LLDPE) geomembrane with 3.0 mm of thickness without stabilizers under weather exposure in Saudi Arabia. After three months of exposure, the geomembrane presented losses higher than 50% in the tensile properties.

A 16-year-old HDPE geomembrane exhumed from a mining facility in a freshwater pond in Argentina and a 6-year-old HDPE geomembrane exhumed from an environmental liner test site in Canada (both samples were 1.5 mm thick) were evaluated using the OIT test by Rowe and Ewais [18]. The exhumed sample, after 16 years of service, presented complete antioxidant depletion. The exhumed sample from the research site performed well, presenting a modest antioxidant depletion. The antioxidant depletion for the research site was faster for the slope location than the bottom location.

Lavoie et al. [19] used thermal analysis to evaluate a 1.0 mm thick HDPE geomembrane exhumed sample from an industrial water pond after up to 2 years of service. Comparing the exhumed sample with a virgin reference sample, the heating rate of 5 °C min^−1^ at the TG analysis noted a different behavior for the exhumed sample, attributed to the reaction time. For the thermomechanical analysis (TMA), the molecular relaxation between 30–65 °C verified for the reference sample was not noted for the exhumed sample. According to the authors, these differences can indicate changes in the polymer morphology.

HDPE geomembranes are usually applied as a pond liner outdoors for the entire service life. This work evaluated a high-density polyethylene smooth geomembrane sample that was 1.0 mm thick, often used for geotechnical, environmental, and agricultural applications, exposed by the Xenon arc weatherometer for 2160 h (90 days) in the short-term of accelerated laboratory exposure, and the ultraviolet fluorescent weatherometer in the short-term, medium-term, and long-term for 8760 h (365 days) of accelerated laboratory exposure to understand the geomembrane’s behavior under ultraviolet exposure. This work compared the geomembrane’s behavior between different ultraviolet devices for short-term exposure, provided new data for the Xenon arc device exposure for the geosynthetics study, and also provided new data for both the DSC analysis and FTIR analysis for HDPE geomembranes after laboratory exposure. The evaluation was performed using the MFI test, OIT tests, tensile test, DSC analysis, and FTIR analysis.

## 2. Materials and Methods

### 2.1. HDPE Geomembrane and Accelerated Weathering Tests

The present work analyzed an HDPE smooth geomembrane with 1.0 mm of nominal thickness manufactured in Brazil. The material was produced by the extrusion-blown film process and formulated with 96–97.5% of medium-density polyethylene (density ≥ 0.940 g cm^−3^), 2–3% of carbon black and 0.5–1.0% of antioxidants and thermostabilizers [20]. The geomembrane’s roll has dimensions of 5.90 m of width and 100 m of length. Table 1 shows the samples’ initial properties.

The GRI-GM13 [32] is an American standard specification for HDPE smooth and textured geomembrane manufacturing quality control. This sample showed a carbon black content slightly higher than the required value range (2–3%), stress crack resistance lower than the minimum required value (500 h), and lower than the OIT minimum value required for the OIT (100 min for the Standard OIT and 400 min for the High Pressure OIT).

A UV-weathering chamber was used from Equilam at Diadema, Brazil, model EQUV, with fluorescent UVA-340 lamps, manufactured by Philips, programmed to work in cycles of 20 ± 0.01 h of UV light at 75 ± 1 °C followed by 4 ± 0.01 h of condensation at 50 ± 0.01 °C for periods of 960, 4380, and 8760 ± 0.01 h. As a reference, the standard test method used was described by ASTM D7238 [33]. Moreover, the Xenon arc chamber used was from Q-Lab in Cleveland, USA, model Xe-3-HS, with UVA-340 fluorescent lamps, manufactured by Philips. The equipment was programmed to operate in 102 ± 0.01 min cycles with a black panel temperature of 60 ± 1 °C followed by 18 ± 0.01 min of light and water spray for periods of 960, 1639, and 2160 h ± 0.01 h. As a reference, the standard test method used was described by ASTM G155 [34].

### 2.2. Melt Flow Index (MFI) Test

The melt flow index test [23] was carried out using a plastometer manufactured by Instron at Norwood, USA, model CEAST MF20. The material was extruded through a smooth bore (2.095 ± 0.005 mm in diameter and 8000 ± 0.025 mm long) at 190 ± 0.08 °C with a 5.0 ± 0.01 kg of deadweight. After 10 ± 0.01 min of the sample extrusion, the material mass was measured using an analytical balance with 0.0001 g of precision.

### 2.3. Tensile Properties

The tensile test [26] was performed using a universal machine, manufactured by EMIC at São José dos Pinhais, Brazil, model DL 3000, equipped with a 2-kN load cell and pneumatic grips. The material was tested only for the machine direction and the analysis was realized for the tensile at break. A test speed of 50 ± 0.05 mm min^−1^ and the IV dog bone specimen were used. This test was conducted only for the aged samples from the UV-weathering chamber. The aged samples from the Xenon arc chamber did not present an adequate size to conduct this test.

### 2.4. Oxidative-Induction Time (OIT) Tests

The oxidative-induction time (OIT) was performed for the standard test (Std. OIT) [30] and high-pressure test (HP OIT) [31], using DSC equipment, model Q20, manufactured by TA Instruments in New Castle, USA, and an aluminum crucible (sample mass of 5 ± 0.5 mg). These tests were conducted in two phases: the endothermic reaction using nitrogen gas purge followed by the specimen oxidation. The standard test was conducted at 200 ± 2 °C with a constant oxygen pressure of 140 ± 5 kPa, a heating rate of 20 ± 1 °C min^−1^ and flow rate of 50 ± 5 mL min^−1^. The high-pressure test was conducted at 150 ± 0.5 °C with a heating rate of 20 ± 1 °C min^−1^ and a constant oxygen pressure of 3.4 ± 0.06 MPa.

### 2.5. Differential Scanning Calorimetry (DSC) Analysis

The analysis was conducted using a nitrogen gas purge with a flow of 50 ± 5 mL min^−1^, an aluminum crucible with a sample mass of 5 ± 0.5 mg, a heating rate of 10 ± 1 °C min^−1^, and a temperature range of 25 to 200 ± 2 °C. The DSC equipment used was manufactured by TA Instruments at New Castle, USA, model Q20.

### 2.6. Fourier Transform Infrared Spectroscopy (FTIR) Analysis

The FTIR analysis was carried out using a spectrometer manufactured by Thermo Scientific at Waltham, USA, model Nicolet iS10, with a wavenumber range of 400–4000 ± 4 cm^−1^. The samples were analyzed in spectra using the attenuated total reflectance (ATR) technique. This technique is used to obtain good quality spectra in solid materials, which must be in perfect contact with the crystal. The conditions for recording the spectra were 16 sweeps and a scan resolution of 4.0 cm^−1^, a sweep speed of 0.20 ± 0.01 cm s^−1^ at 20 ± 1 °C. The spectrum was obtained using the OMNIC program. The absorption bands and peak intensity were obtained using the function “find peaks”.

## 3. Results and Discussion

### 3.1. Melt Flow Index (MFI) Test Results

**Figure 1:**
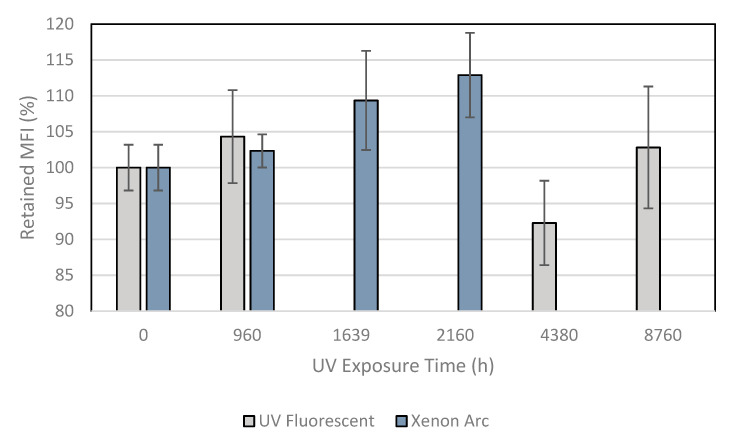
MFI test results showing behavior of the samples after UV exposures.

Table 2 presents the results of the MFI tests after ultraviolet exposure in the laboratory. Figure 1 shows the behavior of the samples in percent for the two laboratory exposure devices retained from the property. It can be observed that for the exposure in the Xenon arc chamber, the sample showed continuous growth in the melt flow index during the exposure times. On the other hand, the sample under exposure by the UV fluorescent device showed variations in the results of the MFI tests during the exposure times.

Many authors [20,35,36] described an understanding of the melt flow index result behavior. Generally, a decrease in MFI test results can indicate an increase in molecular weight by cross-linking reactions; whereas, an increase in MFI can indicate a decrease in molecular weight due to chain scission reactions. The MFI is a qualitative test to assess the polymer’s molecular weight. If the MFI value does not change after exposure, this method is not sufficient to understand the material behavior. In this case, both reactions (cross-linking and chain scission) could occur at the same time. Lodi et al. [20] evaluated the MFI test results of high-density polyethylene geomembranes after 30 months of weathering exposure in Brazil. The results showed an increase of up to 50% for the 2.5 mm-thick sample and decreased to 15% for the 0.8 mm-thick sample. According to the authors, the melt flow index results for the 2.5 mm-thick geomembrane indicates chain scission.

The sample in the Xenon arc exposure chamber showed increases of 2, 9, and 13%, respectively, for 960, 1639 and 2160 h of exposure. The increasing trend can indicate changes in the polymer morphology. However, it was noted that the sample exposed in the UV fluorescent device showed variations between the virgin samples of 4, −8, and 3%, respectively, for 960, 1639, and 2160 h of exposure. In this case, there is no tendency to explain the behavior of the material. These variations are probably related to variation in the material’s properties due to the manufacturing process and variation in exposure to the UV fluorescent device. The standard test method ASTM D7238 [33] prescribes the rotation of the samples in the UV fluorescent device every week to minimize variations among the samples.

### 3.2. Tensile Properties

**Figure 2:**
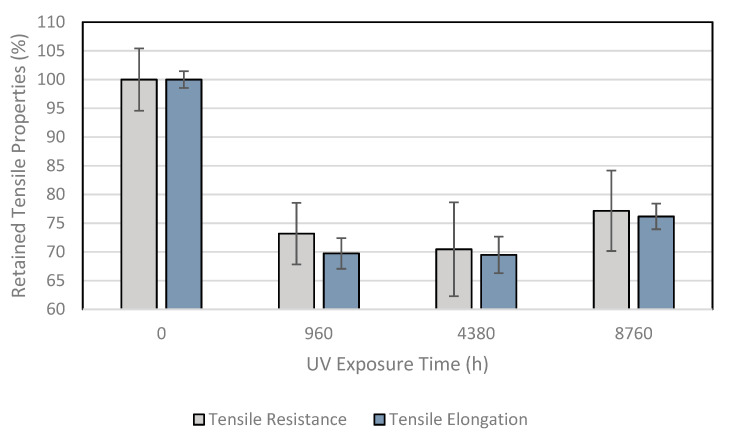
Tensile behavior of the samples after UV fluorescent device exposure.

Table 3 presents the tensile test results at break for the UV fluorescent device exposure. Figure 2 shows the tensile behavior of the sample as a percentage retained from the property. The same behavior was noted for the resistance and elongation properties during the exposure time. The sample exhibited a tendency to lose ductility, without showing brittle behavior.

Tensile properties were evaluated by Noval et al. [37] for an HDPE geomembrane with 1.5 mm of thickness during 20 years of weathering exposure in the San Isidro reservoir in Spain. The resistance and elongation presented the same trend during the time examined. The lowest values found were after 8 years of exposure, with decreases of around 45 and 60%, respectively, for tensile strength and tensile elongation. After 20 years of exposure, the final evaluation showed decreases of about 25 and 35%, respectively, for the tensile resistance and tensile elongation.

Lavoie et al. [19] noted the tensile brittle behavior tendency for a high-density polyethylene geomembrane with 1.0 mm of thickness after 2.25 years of weathering exposure in an industrial water pond in Brazil.

The sample under UV fluorescent device exposure presented decreases for the tensile resistance of 27, 30, and 23%, respectively, for 960, 4380, and 8760 h of exposure. The sample presented decreases for the tensile elongation of 30, 31, and 24%, respectively, for 960, 4380, and 8760 h of exposure. On average, the resistance and elongation decreased during the exposure times by around 30%.

### 3.3. Oxidative-Induction Time (OIT) Test Results

#### 3.3.1. Standard Oxidative-Induction Time (Std. OIT) Test Results

**Figure 3:**
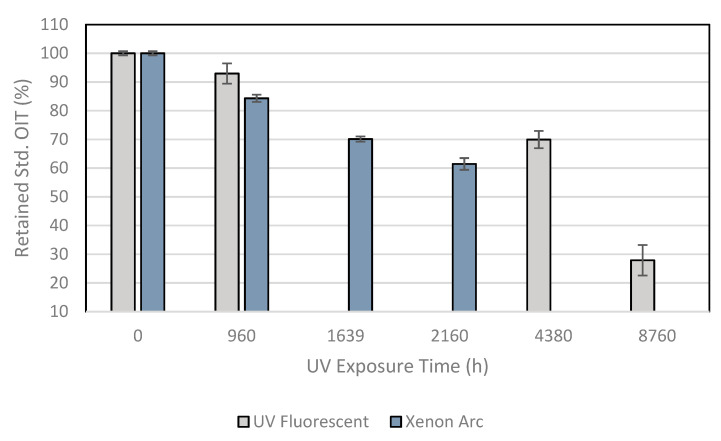
Std. OIT test results showing behavior of the samples after UV exposures.

Table 4 presents the Std. OIT test results after the ultraviolet exposures at the laboratory. Figure 3 shows the behavior of the sample for both laboratory exposure devices in percent retained from the property. The antioxidant depletion for both exposures can be observed. The fastest antioxidant depletion occurred for the Xenon arc exposure chamber considering that the exposure time was shorter than the exposure in the fluorescent UV device.

Reis et al. [14] evaluated the Std. OIT results for two weathering exposure locations in Portugal. After 12 years of weathering exposure, the 2.0 mm-thick geomembrane samples exposed at Bigorne and Viana de Castelo showed decreases of about 40 and 50%, respectively.

The sample under exposure in the Xenon arc exposure chamber showed a decrease in OIT values of 16, 30, and 39%, respectively, for 960, 1639, and 2160 h of exposure. The sample under UV fluorescent device exposure demonstrated decreases of 7, 30, and 72%, respectively, for 960, 4380, and 8760 h of exposure. The comparison of the OIT values between the Xenon arc exposure chamber in 1639 h and the fluorescent UV exposure device in 4380 h showed approximately the same consumption of antioxidants, demonstrating that the fastest depletion rate of antioxidants was for the Xenon arc exposure chamber. The fast antioxidant depletion was noted for both exposure devices, demonstrating the ultraviolet radiation effect on the material’s antioxidants.

#### 3.3.2. High-Pressure Oxidative-Induction Time (HP OIT) Test Results

**Figure 4:**
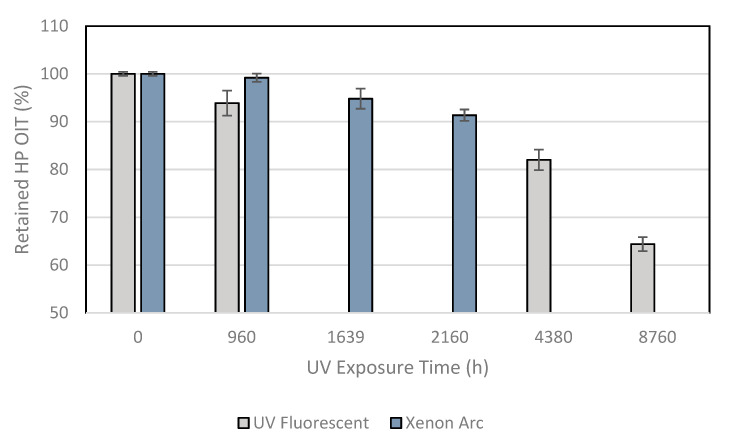
HP OIT test results showing behavior of the samples after UV exposures.

Table 5 presents the HP OIT test results after the ultraviolet exposures in the laboratory. Figure 4 shows the behavior of the sample for the two laboratory exposure devices in percent retained from the property. Similar antioxidant depletion for both device exposures was noted.

According to Hsuan et al. [35], the high-pressure OIT test can distinguish different types of antioxidants, and the test operation’s temperature promotes a good relation to the service conditions.

The sample in the Xenon arc exposure chamber demonstrated decreases of 0.8, 5, and 9%, respectively, for 960, 1639, and 2160 h of exposure. The sample under UV fluorescent device exposure demonstrated decreases of 6, 18, and 36%, respectively, for 960, 4380, and 8760 h of exposure. A lower antioxidant depletion compared with the Std. OIT test was noted for both exposures.

### 3.4. Differential Scanning Calorimetry (DSC) Analysis

**Figure 5:**
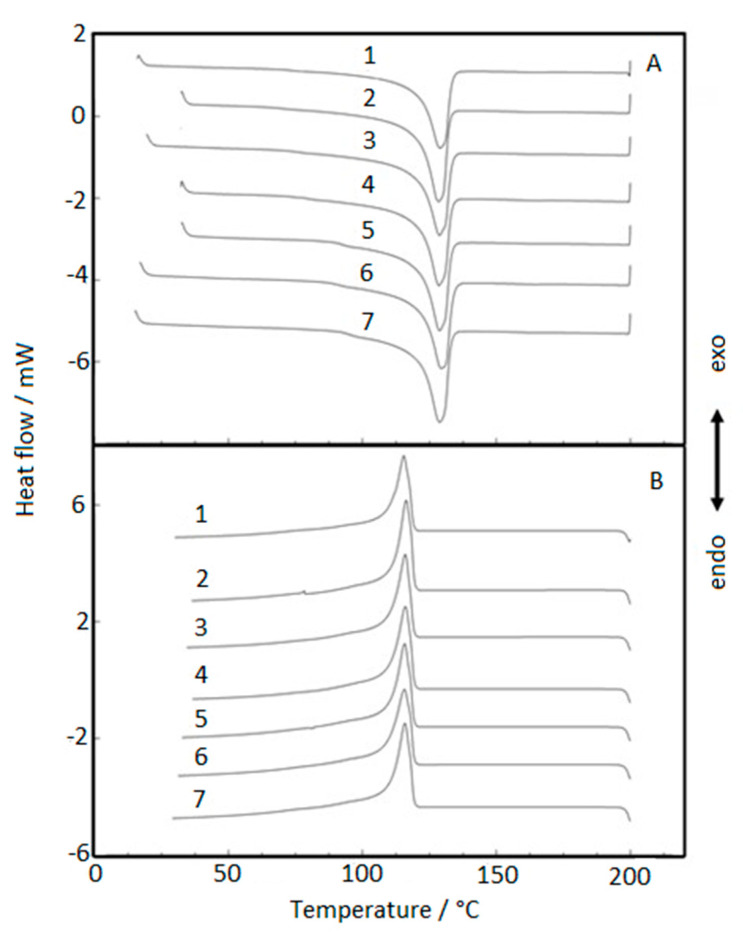
DSC curves: (**A**) melting point stage and (**B**) crystallization stage. 1—virgin sample; 2—960 h Xenon arc sample; 3—1639 h Xenon arc sample; 4—2160 h Xenon arc sample; 5—960 h UV fluorescent sample; 6—4380 h UV Fluorescent sample; and 7—8760 h UV fluorescent sample.

Figure 5 shows the samples’ DSC curves of heating (A) and cooling (B). A curves’ overlap adjustment was performed to coincide with the peaks. Initially, the samples were heated up to 200 °C, where a peak attributed to the melting point was observed (Figure 5A). For all samples, the melting point essentially has the same behavior, without presenting any displacement or overlapping reactions due to the different exposure time of the samples analyzed. This behavior can be explained because the polymer under melting does not present crystallinity, predominating the disordered molecular material. The degraded molecules by ultraviolet radiation or other radiation are involved in the melting process, making it difficult to identify the degradation of the molecules. The crystallization of the different samples can be seen in Figure 5B. The crystallization process tends to ordinate the polymer’s melted molecules, leading to an exothermic process. Thus, the molecules’ degradation was hidden by the molecules’ reorientation, obtaining coincident crystallization points at the DSC curves. The glass transition behavior depends on a cooling system and was not obtained in this work. This analysis is necessary for future evaluations.

### 3.5. Fourier Transform Infrared Spectroscopy (FTIR) Analysis

**Figure 6:**
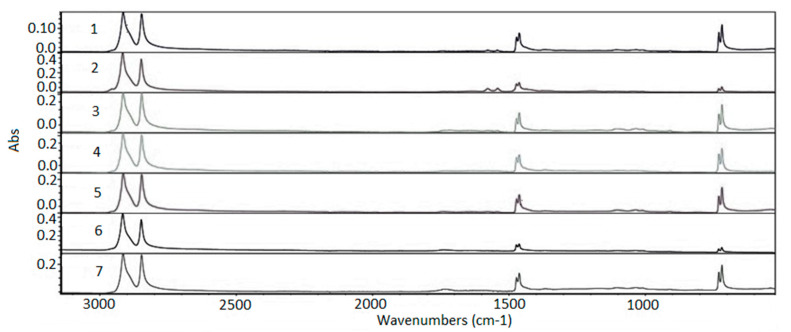
Absorption spectra region from 600 to 3100 cm^−1^. 1—virgin sample; 2—960 h Xenon arc sample; 3—1639 h Xenon arc sample; 4—2160 h Xenon arc sample; 5—960 h UV fluorescent sample; 6—4380 h UV fluorescent sample; and 7—8760 h UV fluorescent sample.

Figure 6 shows the FTIR analysis for the UV fluorescent exposure device and the Xenon arc exposure chamber, highlighting the range from 600 to 3100 cm^−1^ to check the possibility of formations, breaks in the functional groups, and oxidative reactions. The analyzed samples are formed by repetitions of CH_2_ groups. Consequently, the appearance of absorbance bands characteristic of simple C–H bonds and Covalent C–C bonds is expected using this technique [38,39].

**Figure 7:**
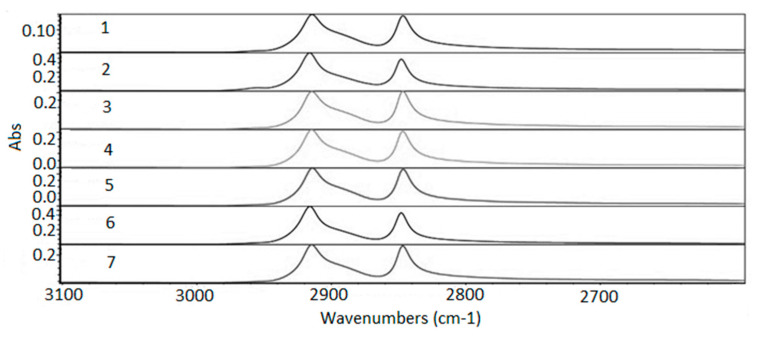
Absorption spectra region from 3100 to 2600 cm^−1^. 1—virgin sample; 2—960 h Xenon arc sample; 3—1639 h Xenon arc sample; 4—2160 h Xenon arc sample; 5—960 h UV fluorescent sample; 6—4380 h UV fluorescent sample; and 7—8760 h UV fluorescent sample.

**Figure 8:**
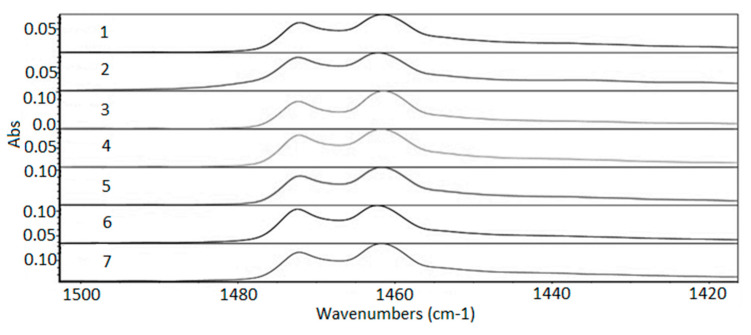
Absorption spectra region from 1420 to 1500 cm^−1^. 1—virgin sample; 2—960 h Xenon arc sample; 3—1639 h Xenon arc sample; 4—2160 h Xenon arc sample; 5—960 h UV fluorescent sample; 6—4380 h UV fluorescent sample; and 7—8760 h UV fluorescent sample.

**Figure 9:**
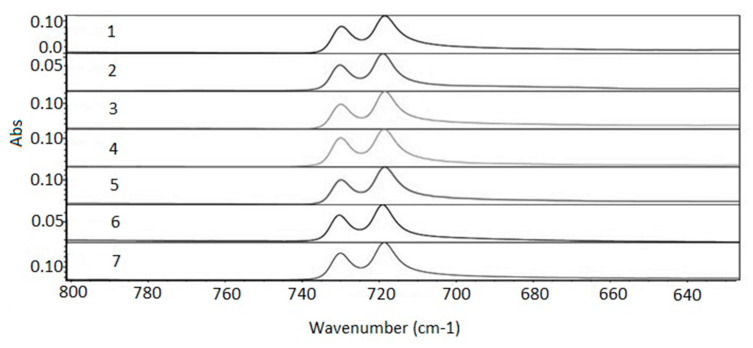
Absorption spectra region from 620 to 800 cm^−1^. 1—virgin sample; 2—960 h Xenon arc sample; 3—1639 h Xenon arc sample; 4—2160 h Xenon arc sample; 5—960 h UV fluorescent sample; 6—4380 h UV fluorescent sample; and 7—8760 h UV fluorescent sample.

**Figure 10:**
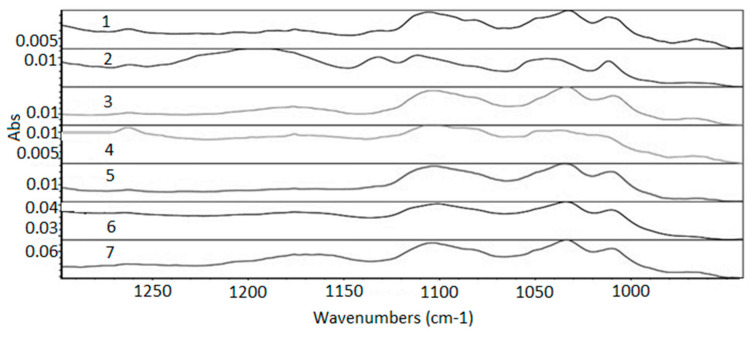
Absorption spectra region from 1300 to 950 cm^−1^. 1—virgin sample; 2—960 h Xenon arc sample; 3—1639 h Xenon arc sample; 4—2160 h Xenon arc sample; 5—960 h UV fluorescent sample; 6—4380 h UV fluorescent sample; and 7—8760 h UV fluorescent sample.

The three modes of the C–H link vibration can be observed through the spectrum. The bands observed in the range of 2800 to 3000 cm^−1^ refer to axial deformations C-H (Figure 7). The angular vibration of CH_2_ was observed in the range of 1400 to 1500 cm^−1^ (Figure 8) and the bands referring to the asymmetric deformation of CH_2_ were noted in the region of 700–730 cm^−1^ (Figure 9) [39]. The low-intensity bands observed in the region between 1000–1250 cm^−1^ refer to the link’s vibrations C–C (Figure 10) [40]. It was not possible to notice a significant variation in the obtained spectra, since the absorption intensities of the bands were close.

It can be observed that all the functional groups characteristic of the polymer were preserved showing that the exposures carried out under different conditions did not cause the departure of any functional group and no reaction was also observed.

Variations in samples’ absolute absorbance values were observed, probably related to the different conditions that samples were exposed to compared with the virgin sample. The degradations caused by the exposures can indicate small morphological changes in the polymers that did not present a trend. However, greater absolute absorbances can be observed in the total spectrum (region of 600–3100 cm^−1^) for the exposed samples compared with the virgin sample. Moynihan [41] also observed changes in the infrared spectra for the PTFE polymer with different morphologies and related this change to the intensity of the peaks. The intensity of the peaks decreased with the increase in crystallinity caused by the exposure of the samples in different degradation conditions. The changes were not sufficient to cause breaks in the functional group or other reactions and did not follow a trend compared with the virgin sample.

## 4. Conclusions

This manuscript analyzed the behavior of 1.0 mm-thick HDPE virgin geomembrane under exposures to two ultraviolet radiation devices for different exposure times. The characterization of the virgin samples showed property values according to the GRI-GM13, with exceptions for stress crack resistance and oxidative induction time values (for standard and high-pressure tests), which showed values lower than the minimum required values. The HDPE geomembrane’s stress crack resistance is led by the resin’s density and molecular weight distribution. The OIT values depend on the quantity and the quality of the geomembrane’s additive package.

The MFI is a qualitative test to assess the polymer’s molecular weight. The sample under Xenon arc exposure chamber showed a trend to increase the MFI’s values during the exposure time. This increasing trend can indicate changes in the polymer’s morphology. The sample under UV fluorescent device exposure presented variations among the virgin sample without a trend.

The tensile behavior analysis showed a sample’s trend to lose its ductileness, without presenting brittle behavior. The resistance and elongation decrease during the exposure time for the UV fluorescent device exposure was, on average, about 30%.

The samples’ OIT test results under both device exposures showed faster antioxidant depletion for the standard OIT test than the high-pressure OIT test. The higher temperature of the standard assay may have contributed to the faster antioxidant depletion. After 8760 h of exposure, the sample under UV fluorescent device exposure decreased 75% for the Std. OIT test and 36% for the HP OIT test. For the Xenon arc exposure chamber, the sample tested demonstrated a decrease of 35% for the Std. OIT test and 8% for the HP OIT test after 2160 h of exposure. For the Std. OIT test results, considering the lower exposure time, the fastest antioxidant depletion occurred for the Xenon arc exposure chamber than the UV fluorescent device exposure.

The samples’ DSC curves under heating and cooling presented no differences during the exposure for both devices. The coincident melting and crystallization points obtained can be explained by the predominating disordered molecular material in the endothermic process and the trend to ordinate the polymer’s melted molecules in the exothermic process.

The FTIR analysis was carried out for the range of 600 to 4000 cm^−1^ to check for possible formations, breaks in functional groups, and oxidative reactions. For both exposures the analysis showed that it did not cause any departure from functional groups and no reaction was observed. All functional groups characteristic of the polymer were preserved. Variations in samples’ absolute absorbance values were observed, probably related to the different conditions that samples were exposed to compared with the virgin sample. The changes were not sufficient to cause breaks in the functional group or other reactions and did not follow a trend compared with the virgin sample.

## Figures and Tables

**Table 1:** Initial values of high-density polyethylene geomembrane’s properties.

Property	Method	Unit	Mean Value
Thickness	ASTM D5199 [21]	mm	1.040 (±0.079)
Density	ASTM D792 [22]	g cm^−3^	0.958 (±0.002)
MFI (5.0 kg/190 °C)	ASTM D1238 [23]	g 10 min^−1^	0.6800 (±0.0217)
Carbon black content	ASTM D4218 [24]	%	3.14 (±0.13)
Carbon black dispersion	ASTM D5593 [25]	Category	Category I
Tensile break resistance	ASTM D6693 [26]	kN m^−1^	28.52 (±1.55)
Tensile break elongation		%	733.47 (±10.76)
Tear resistance	ASTM D1004 [27]	N	169.17 (±8.76)
Puncture resistance	ASTM D4833 [28]	N	434.17 (±18.19)
Stress crack resistance	ASTM D5397 [29]	h	16.47 (±2.66)
Standard OIT	ASTM D3895 [30]	min	40.49 (±0.73)
High-pressure OIT	ASTM D5885 [31]	min	125.70 (±0.42)

Standard deviations are between brackets.

**Table 2:** MFI test results after UV laboratory exposures.

Sample	Exposure Time (h)	MFI (g min^−1^)	MFI (%)
Virgin	0	0.6800 (±0.0217)	100.0
UV fluorescent	960	0.7094 (±0.0460)	104.33
UV fluorescent	4380	0.6275 (±0.0369)	92.29
UV fluorescent	8760	0.6991 (±0.0594)	102.81
Xenon arc	960	0.6958 (±0.0161)	102.33
Xenon arc	1639	0.7437 (±0.0514)	109.36
Xenon arc	2160	0.7677 (±0.0453)	112.89

Standard deviations are between brackets.

**Table 3:** Tensile at break test results after UV fluorescent device exposure.

Exposure Time (h)	Tensile Resist. (kN m^−1^)	Tensile Resist. (%)	Tensile Elong. (%)	Tensile Elong. (%)
0	28.52 (±1.55)	100.0	733.47 (±10.76)	100.0
960	20.87 (±1.12)	73.18	511.47 (±13.67)	69.73
4380	20.09 (±1.64)	70.45	509.67 (±16.20)	69.49
8760	20.00 (±1.54)	77.15	558.70 (±12.47)	76.17

Standard deviations are between brackets.

**Table 4:** Std. OIT test results after the UV laboratory exposures.

Sample	Exposure Time (h)	Std. OIT (min)	Std. OIT (%)
Virgin	0	40.49 (±0.73)	100
UV fluorescent	960	37.63 (±3.30)	92.94
UV fluorescent	4380	28.32 (±2.11)	69.93
UV fluorescent	8760	11.30 ((±1.48)	27.91
Xenon arc	960	34.15 (±1.27)	84.33
Xenon arc	1639	28.40 (±0.93)	70.13
Xenon arc	2160	24.88 (±2.04)	61,45

Standard deviations between brackets.

**Table 5:** HP OIT test results after UV laboratory exposures.

Sample	Exposure Time (h)	HP OIT (min)	HP OIT (%)
Virgin	0	125.70 (±0.42)	100
UV fluorescent	960	118.02 (±2.62)	93.89
UV fluorescent	4380	103.09 (±2.13)	82.01
UV fluorescent	8760	80.96 (±1.46)	64.40
Xenon arc	960	124.69 (±0.86)	99.20
Xenon arc	1639	119.17 (±2.10)	94.80
Xenon arc	2160	114.83 (±1.20)	91.35

Standard deviations are between brackets.

## References

[1] Koerner R.M. (2005). Designing with Geosynthetics.

[2] Rollin A.R., Rigo J.M. (1991). Geomembranes—Identification and Performance Testing.

[3] Vertematti J.C. (2015). Manual Brasileiro de Geossintéticos.

[4] Rowe R.K., Sangam H.P. (2002). Durability of HDPE Geomembranes. Geotext Geomembr..

[5] Koerner G.R., Hsuan Y.G., Koerner R.M., Sarsby R.W. (2007). The durability of geosynthetics. Geosynthetics in Civil Engineering.

[6] Kelen T. (1983). Polymer Degradation.

[7] Lavoie F.L., Kobelnik M., Valentin C.A., da Silva J.L. (2020). Durability of HDPE geomembranes: An overview. Quim. Nova.

[8] Van Santvoort G. (1994). Geotextiles and Geomembranes in Civil. Engineering.

[9] Sharma H.D., Lewis S.P. (1994). Waste Containment System, Waste Stabilization and Landfills: Design and Evaluation.

[10] Suits L.D., Hsuan Y.G. (2003). Assessing the photo-degradation of geosynthetics by outdoor exposure and laboratory weatherometer. Geotext. Geomembr..

[11] Koerner R.M., Hsuan Y.G., Koerner G.R. Freshwater and geosynthetics; A perfect marriage. Proceedings of the 1st Pan American Conference on Geosynthetics.

[12] Sahu A.K., Sudhakar K., Sarviya R.M. (2019). Influence of U.V light on the thermal properties of HDPE/Carbon black composites. Case Stud. Therm. Eng..

[13] Jiang T., Qi Y., Wu Y., Zhang J. (2019). Application of antioxidant and ultraviolet absorber into HDPE: Enhanced resistance to UV irradiation. e-Polymers.

[14] Reis R.K., Barroso M., Lopes M.G. (2017). Evolução de cinco geomembranas expostas a condições climáticas em Portugal durante 12 anos. Geotecnia.

[15] Martínez-Romo A., González-Mota R., Soto-Bernal J.J., Rosales-Candelas I. (2015). Investigating the Degradability of HDPE, LDPE, PE-BIO, and PE-OXO Films under UV-B Radiation. J. Spectrosc..

[16] Mendes L.C., Rufino E.S., de Paula F.O.C., Torres A.C. (2003). Mechanical, thermal and microstructure evaluation of HDPE after weathering in Rio de Janeiro City. Polym. Degrad. Stab..

[17] Qureshi F.S., Amin M.B., Maadha A.G., Hamid S.H. (1989). Weather-induced degradation of linear low-density polyethylene: Mechanical properties. Polym.-Plast. Technol. Eng..

[18] Rowe R.K., Ewais A.M.R. (2015). Ageing of exposed geomembranes at locations with different climatological conditions. Can. Geotech. J..

[19] Lavoie F.L., Valentin C.A., Kobelnik M., Lins da Silva J., Lopes M.L. (2020). Study of an exhumed HDPE geomembrane used in an industrial water pond: Physical and thermoanalytical characterisations. Results Mater..

[20] Lodi P.C., Bueno B.S., Vilar O.M. (2013). The effects of weathering exposure on the physical, mechanical, and thermal properties of high-density polyethylene and poly (vinyl chloride). Mater. Res..

[21] ASTM (American Society for Testing and Materials) (2019). ASTM D5199 Standard Test Methods for Measuring the Nominal Thickness of Geosynthetics.

[22] ASTM (American Society for Testing and Materials) (2020). ASTM D792 Standard Test Methods for Density and Specific Gravity (Relative Density) of Plastics by Displacement.

[23] ASTM (American Society for Testing and Materials) (2020). ASTM D1238 Standard Test Methods for Melt Flow Rates of Thermoplastics by Extrusion Plastometer.

[24] ASTM (American Society for Testing and Materials) (2020). ASTM D4218 Standard Test Method for Determination of Carbon Black Content in Polyethylene Compounds by the Muffle-Furnace Technique.

[25] ASTM (American Society for Testing and Materials) (2016). ASTM D5596 Standard Test Method for Microscopic Evaluation of the Dispersion of Carbon Black in Polyolefin Geosynthetics.

[26] ASTM (American Society for Testing and Materials) (2020). ASTM D6693 Standard Test Methods for Determining Tensile Properties of Nonreinforced Polyethylene and Nonreinforced Flexible Polypropylene Geomembranes.

[27] ASTM (American Society for Testing and Materials) (2021). ASTM D1004 Standard Test Methods for Tear Resistance (Graves Tear) of Plastic Film and Sheeting.

[28] ASTM (American Society for Testing and Materials) (2020). ASTM D4833 Standard Test Method for Index Puncture Resistance of Geomembranes and Related Products.

[29] ASTM (American Society for Testing and Materials) (2020). ASTM D5397 Standard Test Method for Evaluation of Stress Crack Resistance of Polyolefin Geomembranes Using Notched Constant Tensile Load Test..

[30] ASTM (American Society for Testing and Materials) (2019). ASTM D3895 Standard Test Method for Oxidative-Induction Time of Polyolefins by Differential Scanning Calorimetry.

[31] ASTM (American Society for Testing and Materials) (2020). ASTM D5885 Standard Test Method for Oxidative Induction Time of Polyolefin Geosynthetics by High-Pressure Differential Scanning Calorimetry.

[32] GRI (Geosynthetic Research Institute) (2021). GRI-GM13 Test Methods, Test Properties and Testing Frequency for High. Density Polyethylene (HDPE) Smooth and Textured Geomembranes.

[33] ASTM (American Society for Testing and Materials) (2020). ASTM D7238 Standard Test Method for Effect of Exposure of Unreinforced Polyolefin Geomembrane Using Fluorescent UV Condensation Apparatus.

[34] ASTM (American Society for Testing and Materials) (2013). ASTM G155 Standard Practice for Operating Xenon Arc Light Apparatus for Exposure of Non-Metallic Materials.

[35] Hsuan Y.G., Koerner R.M. (1998). Antioxidant depletion lifetime in high density polyethylene geomembranes. J. Geotech. Geoenviron. Eng..

[36] Rowe R.K., Islam M.Z., Hsuan Y.G. (2008). Leachate chemical composition effects on OIT depletion in an HDPE geomembranes. Geosynth. Int..

[37] Noval A.M., Blanco M., Castillo F., Leiro A., Mateo B., Zornberg J.G., Aguiar E., Torregrosa J.B., Redón M. Long-term performance of the HDPE geomembrane at the “San Isidro” reservoir. Proceedings of the 10th International Conference on Geosynthetics.

[38] Colthup N.B., Daly L.H., Wiberley S.E. (1990). Introduction to Infrared and Raman Spectroscopy.

[39] Koenig J.L. (1999). Spectroscopy of Polymers.

[40] Stuart B. (2004). Infrared Spectroscopy Fundamentals and Applications.

[41] Moynihan R. (1959). The molecular structure of perfluorocarbon polymers. Infrared studies on polytetrafluoroethylene. J. Am. Chem. Soc..

